# The Effect of a Global Health Shock on Preventive Drug Intake: Evidence from SHARE 2020–2022 for 15 European Countries

**DOI:** 10.21203/rs.3.rs-10795123/v1

**Published:** 2026-08-25

**Authors:** Hans Gevers

**Affiliations:** Estonian Business School, Department Research, Development & PhD Studies

**Keywords:** health shock, preventive drug intake, Europe, SHARE, longitudinal

## Abstract

Self-medication is known to have potential harmful consequences. In times of a health shock, like the COVID-19 pandemic, preventive drug intake may imply irresponsible behaviour. To provide insight into this phenomenon, this study explores the preventive behaviour of 4,642 respondents of the Survey of Health, Ageing and Retirement in Europe (SHARE) for the period 2020–2022 and 15 European countries. It aims at highlighting relevant drivers and relies on an econometric analysis backed by random-effects panel logistic as well as pooled logistic estimators. The results indicate that being female, more educated, with income, in worse health, or out of work due to COVID-19 relates to higher odds of using preventive drugs for COVID-19 disease. Interaction effects illustrate that those who report symptoms are additionally more likely to take preventive drugs when in poor health, with income, or having hardly any difficulties with making ends meet. Surprisingly, preventive drug intake when reporting symptoms decreases at older ages. No relevant impact is found for relationship status, financial distress, and country of citizenship. Overall, the study identifies important drivers of preventive drug intake informative for policymaking in the context of a health shock like COVID-19.

## Introduction

This study aims at providing insight into the drivers of preventive drug intake resulting from a health shock in order to contribute to the research domain of health services usage. It explores which characteristics accompany citizens who have access to drug supply to counter health risks in times of a health shock. This study relies on a quantitative econometric analysis nurtured by high-quality data from SHARE, and identifies which drivers impact preventive drug intake and in which direction. The study can be framed in the theory of Andersen (1968) regarding the Behavioral Model of Health Services Use as it focuses on explaining the use of medical care services. Andersen’s model consists of four components, i.e. the predisposing, enabling, need, and use components. The predisposing component may regard variables covering age, gender, education, and the like. The enabling component can include financial aspects, e.g., income, insurance, and the like. The need component aligns with a given health dimension, e.g., self-perceived health status, a specific disease, and the like (Babitsch et al., 2012). The resultant component is the actual use of health services. In this study, the latter is the use or intake of drugs as a prevention against COVID-19 disease. Furthermore, all three remaining components are accounted for, with, in particular, a focus on their interrelationship with the reporting of symptoms, being a need component. The following sections cover the literature review, data, methodology, results, discussion, and conclusion.

## Literature Review

The existing literature is explored for studies related to self-medication as it reflects the individual explicit act of preventive drug intake at the occurrence of a health shock. In this study, drug intake as advised by health workers or medical institutions is not excluded, yet not discussed in the literature review as it relates to the specific field of prescribed drugs and obeys different mechanisms than self-medication. Additionally, the review documents findings from across the world given the magnitude of COVID-19 and in order to provide an inclusive context for the phenomenon of self-medication.

People across the globe may be individually driven to self-medicate during a health shock like COVID-19, while balancing the risks it accompanies. Several studies elaborate on the occurrence and knowledge of self-medication. Pimentel and de Souza (2024) find high levels of self-reported knowledge regarding self-medication during the pandemic in their sample of Brazilian students, yet this self-reported knowledge appears overstated and, in reality, insufficient. Pelullo, Lombardi, Polla, Napolitano, and Giusepp (2025) find that 67 per cent of their Italian sample know about self-medication and adverse drug reaction. University education appears to boost this knowledge. The sample reports 29 per cent of respondents to self-medicate to prevent COVID-19 disease. In Bangladesh, 15 per cent of the inquired report taking medicines, roughly 60 per cent taking herbal products, while eleven per cent combine both as a preventive practice (Ahmed et al., 2020). A cohort of similar size occurs among Indian medical students, as 29 per cent of them report self-medication as a preventive measure (Pawar et al., 2023). A more diversified Indian sample estimates that 47 per cent rely on self-medication to prevent COVID-19, which is modest relative to the 63 per cent of respondents confirming self-medication during the pandemic as reported by a survey held among citizens of Mogadishu in Somalia (Agrawal et al., 2023; Moussa et al., 2023). An important facilitator of self-medication may be online drug availability. In the United Arab Emirates, the increased likelihood of purchasing medication online during the pandemic can be explained by higher education, relationship status, and gender. Moreover, this purchasing behaviour comes with a risk arising from self-diagnosis, non-alignment with the patienťs medical record, inappropriate mix with other drugs, and the like (Jairoun et al., 2021). Additionally, the self-medication driven by COVID-19 disease may originate from reporting symptoms, or preventing infection. Fear of quarantine, affordability, or convenience potentially influences these motivations (Quincho-Lopez et al., 2021). However, an expected boost in self-medication relatable to the COVID-19 pandemic ought not to be taken for granted, as illustrated by Dare et al. (2022). Their sample of Ugandans reveals a decrease from 88 per cent before to 57 per cent during the pandemic. Moreover, a boost may not result from increased self-medication to counter COVID-19 disease itself, but from reduced medical assistance resulting from the lockdown. In Pakistan, where digital medical assistance is less evident, almost 21 per cent of the inquired citizens acknowledge the relevance of this driver for self-medication (Arain et al., 2021). To conclude, self-medication behaviour is not solely relatable to the occurrence of a health shock, yet is exposed to many other influences.

On top of the individual drivers, the extrinsic drivers ought to be acknowledged as they hold a broad societal risk. The preparedness of a community is relevant in this regard. Mitjà (2020) stresses the need for strategy development to prevent illness and protect people during times of health shocks, like the SARS CoV-2 attacks. On top, without a strategy, the population remains unprotected against the risks of self-medication. Such a risk may be poisoning. Hashemi, Ghareghani, Nasimi and Shahbazi (2023) highlight this risk, yet find the bad effects of overconsumption of drugs, herbal medicines, and the like insufficiently documented. Also Mahmoudi (2022) acknowledges the negative side effects for his Iranian sample. He highlights that drug resistance may arise from the use of antibiotics through self-medicating, and stresses the importance of education and constraining drug availability without prescription. Similarly, intervention is desired in many other countries. Akintunde et al. (2022) promote for the Sub-Saharan Africans self-medication as a solution to reduce the risk of an infection like triggered by the COVID-19 virus. In contrast, in a country like Peru, where 50 per cent of the people self-medicate in an irresponsible way, the COVID-19 pandemic implied not only an increase in this percentage yet also an increase in the risk for adverse effects (Benites-Meza et al., 2022). Irresponsible self-medication may in particular be found among groups with high levels of knowledge, like the Columbian medical student inquired during COVID-19 by Martínez-De La Rosa et al. (2023). Overall, focused interventions may constrain the practice of irresponsible self-medication behaviour.

### Data

To explore preventive drug intake, the output of the recurring health survey SHARE is used (Bergmann et al., 2024; Börsch-Supan et al., 2013). The latter is realised by the European Research Infrastructure Consortium (ERIC) and provides data collected during the COVID-19 pandemic (SHARE-ERIC, 2024a, 2024b). Table 1 on the following page describes the data used.

Table 1 refers to 12 variables, of which nine are considered being categorical. The variable Preventive drug intake COVID-19 is the dependent variable and provides a yes-no answer of the respondent indicating if he or she used drugs or medicine as a prevention against Corona, i.e. the COVID-19 disease. The variable may cover more than self-medication given that a respondent may have sought medical advice regarding preventive drugs. The dependent variable represents the theoretical Use component. Furthermore, the Predisposing component consists of three categorical as well as two continuous variables. The variable 0 Single or 1 Couple indicates the relationship status, while Gender is 1 Male and 2 Female. Out of work due to COVID-19 covers 0 Not applicable, 1 No, and 2 Yes. This variable explicitly results from the shutdown not the disease, as it reports if the respondent became unemployed, was laid off or had to close his or her business. The baseline is not informative, yet included to safeguard observations. Merely the difference between the No and Yes categories is interpreted for this variable. Additionally, Age reveals the average respondent being 70 years old, within a range from 41 to 100 years old. Years of education goes from zero to 25, with an average of 12 years of education. On top of these variables, as part of the Predisposing component, the Need component is specified. Self-perceived health status represents the status before the outbreak of COVID-19 disease, and labels 1 Excellent to 5 Poor. Reporting symptoms indicates if the respondent reports 0 No symptoms or 1 Symptoms. Lastly, the third component, i.e. the Enabling one, consists of two variables. Financial distress indicates whether the respondent has difficulties with making ends meet. It holds four categories, i.e. 1 With great difficulty, 2 With some difficulty, 3 Fairly easily, and 4 Easily. The financial status itself is reflected by 0 Without income or 1 With income, which categorises the respondent’s lowest overall monthly household income since the outbreak. The two remaining variables indicate a geographic and time dimension. As such, the Country of citizenship may be Germany, Sweden, the Netherlands, Spain, Italy, France, Denmark, Switzerland, Belgium, the Czech Republic, Luxembourg, Portugal, Slovenia, Estonia, or Finland, while the Survey year may be 2020 or 2022. The latter is preferably integrated in the estimators as a panel-level variable on top of the respondent’s unique identification number. The compatibility of all variables is tested through the Spearman’s rank correlation listed in Table 3 in Appendix. The dependent variable appears well correlated with the Country (0.147) and Age (−0.117). All other correlations are lower.

**Figure 1:**
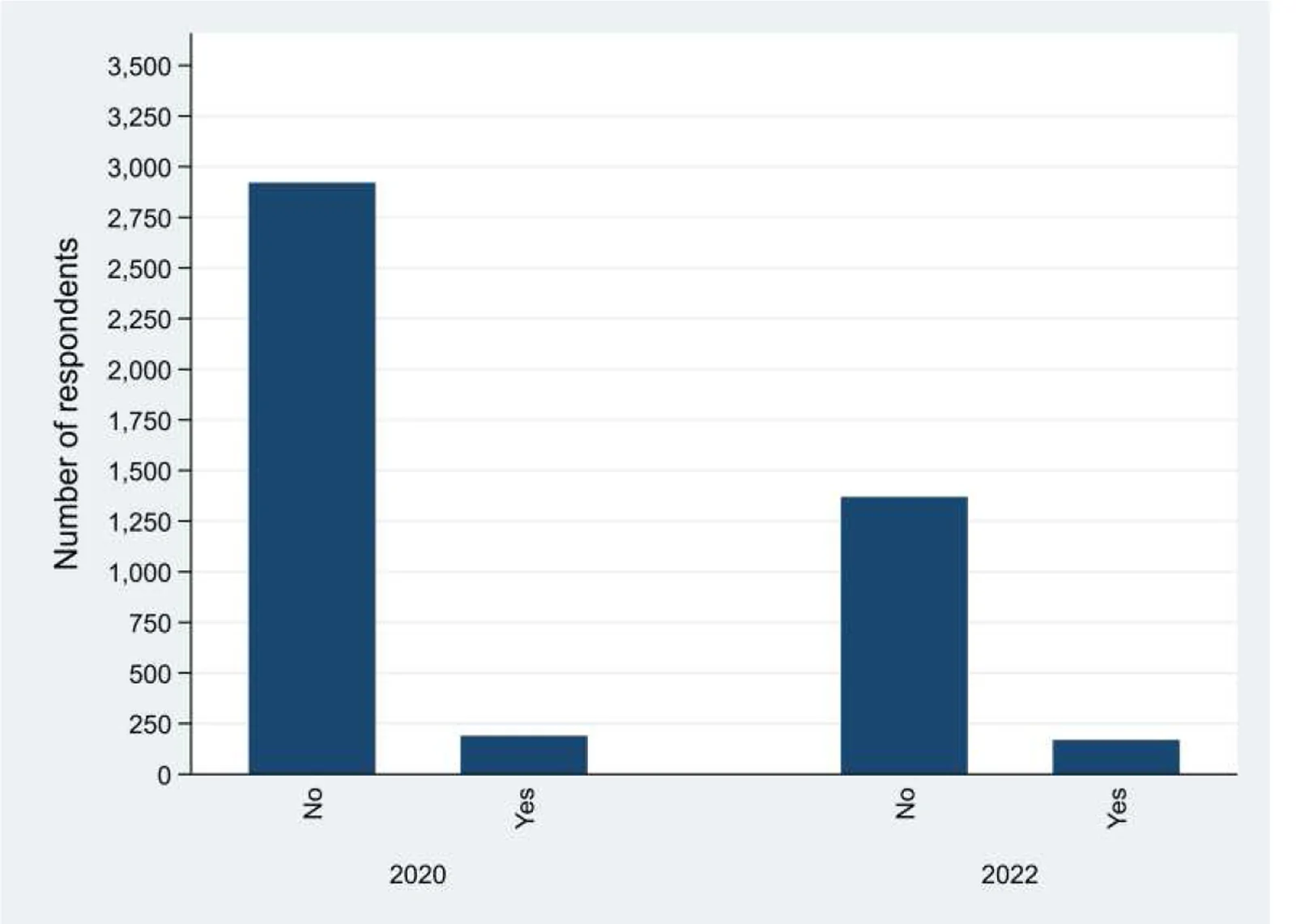
Number of Respondents for Preventive drug intake and per Survey year

**Figure 2:**
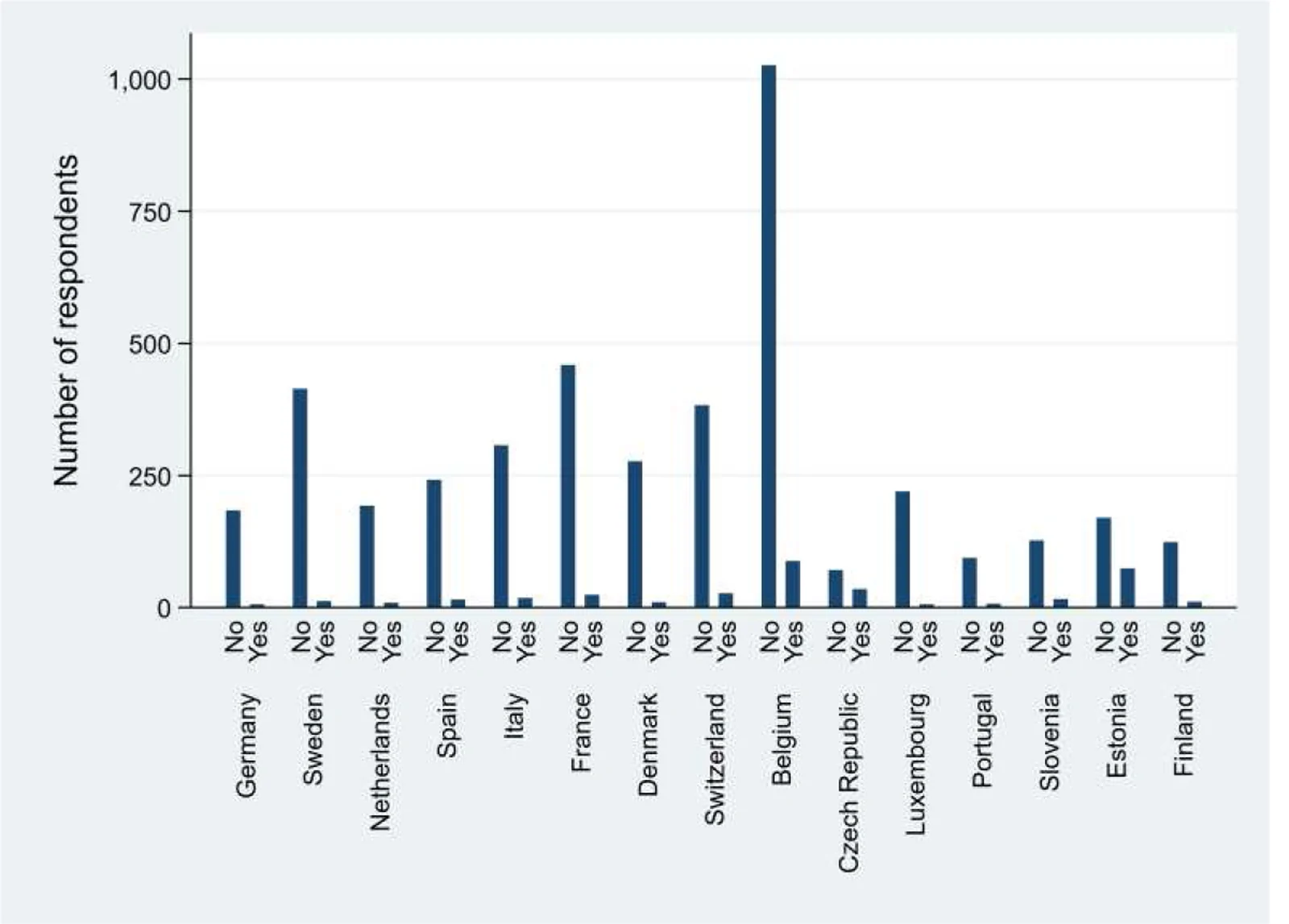
Number of Respondents for Preventive drug intake and per Country

**Figure 3:**
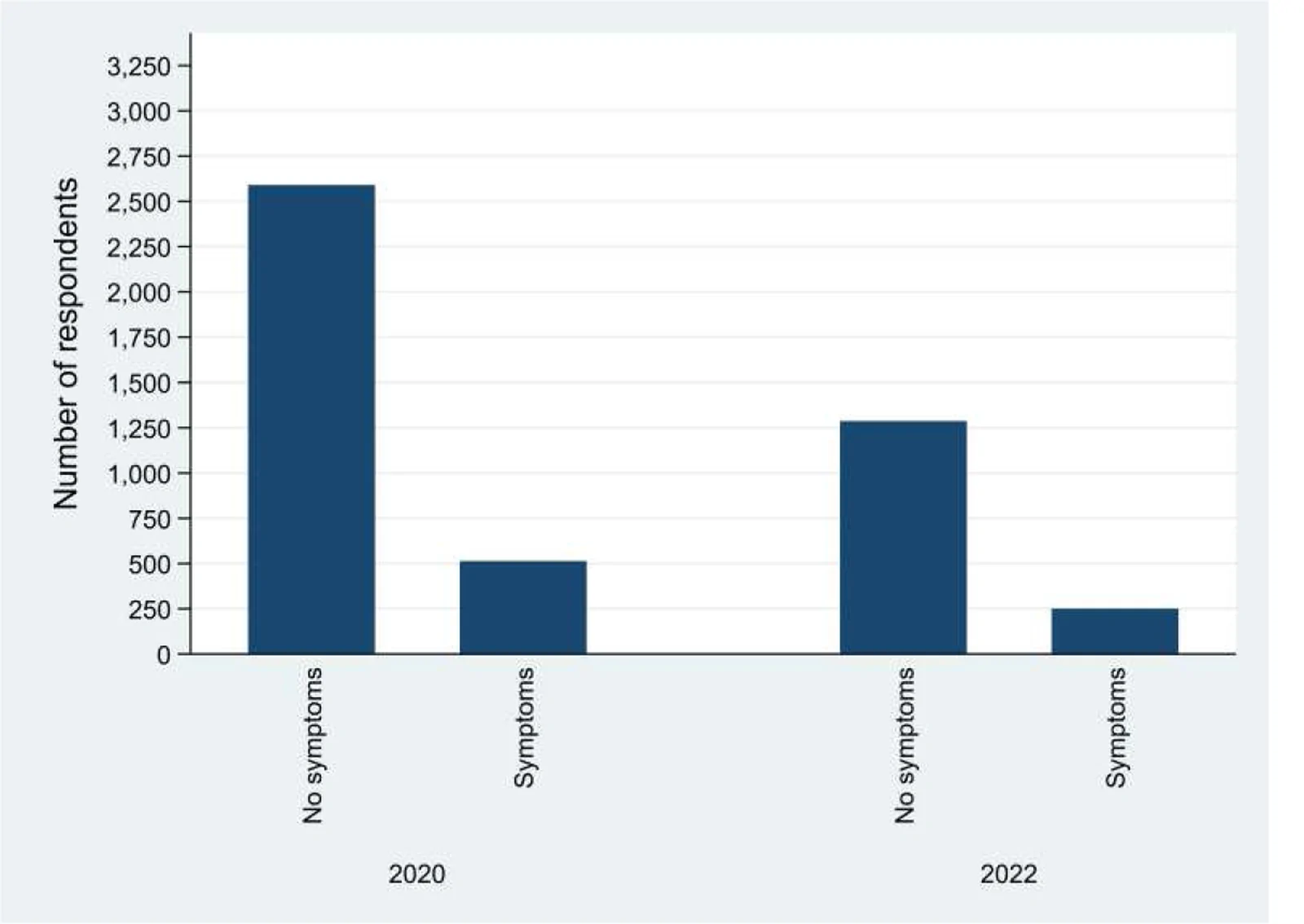
Number of Respondents for Reporting symptoms and per Survey year

**Figure 4:**
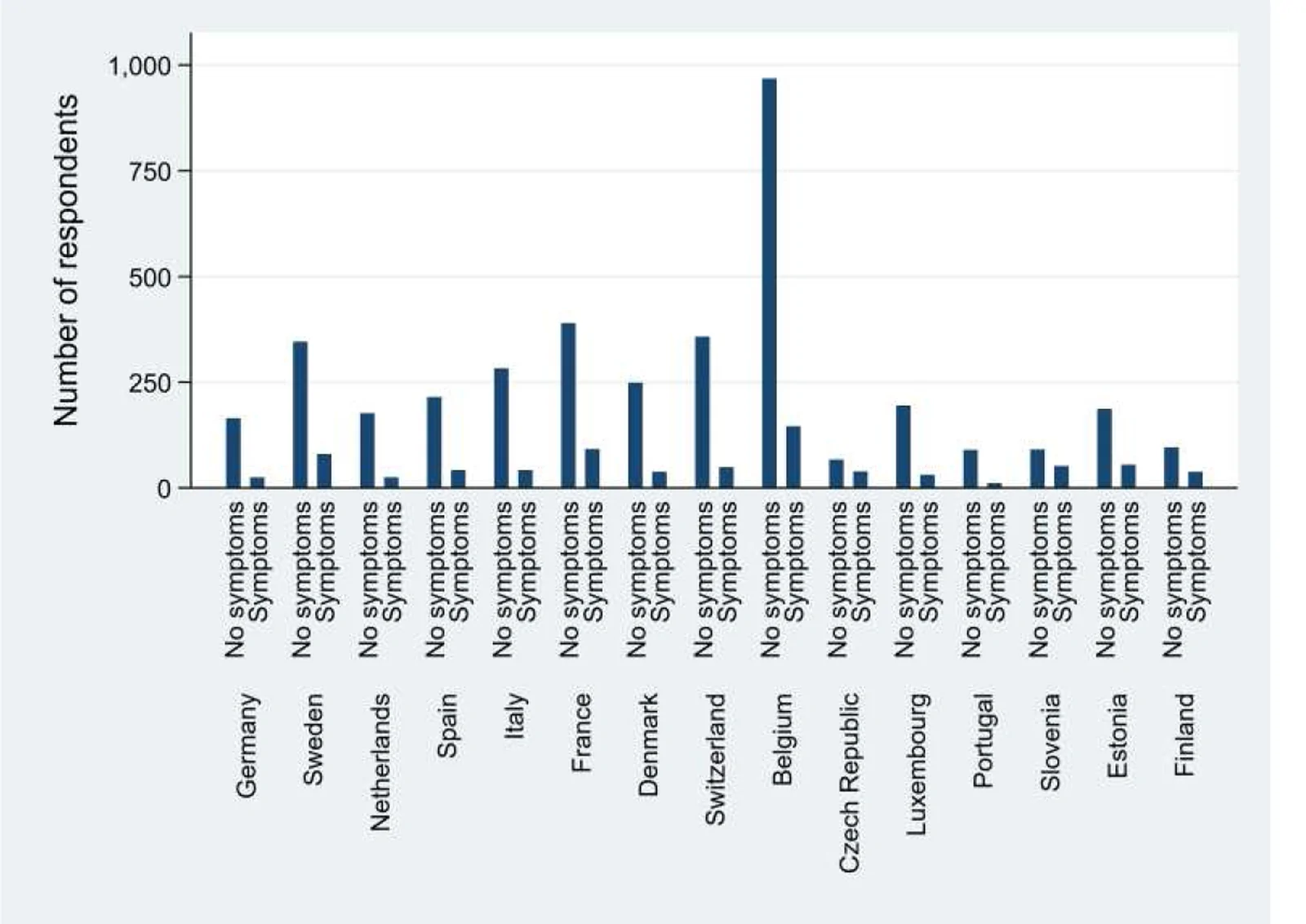
Number of Respondents for Reporting symptoms and per Country

To additionally visually describe several variables of interest, Figures 1 to 4 are included.

Figure 1 shows that the majority of respondents did not take preventive drugs during the period 2020–2022. The figure also indicates that the dataset is unbalanced. It is assumed that neither this imbalance nor the short time horizon troubles the estimators used.

Figure 2 reveals the same as Figure 1, yet per Country. Evidently, respondents still predominantly report no preventive drug intake. The large number of respondents for Belgium draws attention, yet is not considered an issue for the estimation procedure.

Figure 3 illustrates the reporting of symptoms among the respondents. In particular, this variable is of interest as, despite their low correlation (0.089, see Table 3 in Appendix), reporting symptoms is the most sound reason for starting medication. It is, as a Need component, targeted for its impact on the Use component, i.e. preventive drug intake, as well as for its interrelatedness with the Predisposing and Enabling components.

Figure 4 reveals the same as Figure 3, yet per Country. The high number of respondents for Belgium detected in Figure 2, reappears in Figure 4. Furthermore, once again, the majority of the respondents report no symptoms.

A concluding remark regards the prevalence of vaccination among the scrutinised countries. The fact that a respondent uses preventive drugs may be influenced by differences in the vaccination schedules of the targeted countries, yet these schedules are quite similar as illustrated by their governmental agencies (ECDC, s.d.; FOPH, s.d.).

## Methodology

The econometric software Stata 19.5 Basic Edition is deployed to complete the analysis. A total of six logistic estimators are used to document the analysis. Two estimators, being a fixed- and random-effects estimator, are combined to realise the Hausman test (1978). Despite the fact that the usage of the test with logistic panel estimators desires additional theoretical support, its outcome is regarded as an indication that the differences between the fixed- and random-effects estimation procedures are not systematic (Chi-squared 16.896, p=.154, see Materials and Code availability). Yet note that the fixed- and random-effects estimators list contrasting results, as documented in Table 4 in Appendix, implying that questioning the accuracy of the Hausman test in the context of logistic panel estimators is appropriate. To reduce potential bias, the random-effects logistic panel estimator is used in combination with the pooled logistic estimator as a benchmark. The former is documented without calibrated longitudinal weights, and with as well as without interaction effects. The latter is documented, with interaction effects, and with as well as without calibrated longitudinal weights. The latter weights are provided by SHARE and rely on Eurostat data for mortality per year, country, age and gender. Their integration facilitates tackling issues resulting from nonresponse, refreshment, as well as attrition (De Luca & Rossetti, 2018; Pacifico, 2014).

## Results

The results of the two random-effects logistic panel estimators and two logistic pooled estimators are summarised in Table 2 on the following page. The results hold on average and ceteris paribus.

Table 2 reveals the results of the logistic regressions. A priori, estimators (1) and (4) are expected to be most informative, given that estimator (2) includes several superfluous yet illustrative non-significant interaction effects and estimator (3) does not integrate calibrated longitudinal weights. To ensure robust concluding, results are interpreted across estimators. First, the variable of interest, Reporting symptoms, reports a higher odds of using preventive drugs for COVID-19 disease when the respondent reports having symptoms. The odds of estimators (2) and (3) are extreme, acknowledging that they are the less favoured estimators. The odds of estimator (4) are insignificant, yet the main effect of Reporting symptoms is expected to be absorbed by its interactions with income and health. The variable Relationship status may suggest a slightly lower odds of preventive drug intake, yet the effect is not significant at a ten per cent threshold. For Gender, females appear to more likely take drugs preventively compared to males. The effect occurs regardless of having symptoms or not. The variable Age reports non-significant odds across estimators, yet its impact is likely absorbed by its interaction with Symptoms. As such, increasing age when reporting symptoms relates to lower odds of using preventive drugs for COVID-19 disease. In contrast, the main effect, not its interaction with symptoms, reports an increased likelihood of using preventive drugs. Similarly, being out of work due to COVID-19, regardless of having symptoms or not, results in a higher likelihood of using preventive drugs relative to those who are not out of work. The effect occurs regardless of having symptoms or not. The odds are interpretable relative to the category Not out of work, not the baseline. Furthermore, the variable Income suggests that those With income tend to use preventive drugs for COVID-19 disease less likely. It is expectable that those with income are not out of work, implying that the effect contrasts with the impact of being out of work. Interestingly, estimator (4) reports higher odds of using preventive drugs for those with income as well as with symptoms. Plausibly, this interaction effect absorbs the main, for this estimator non-significant, effect of Symptoms. The occurrence of the effect may be driven by people with income, likely at work, who report symptoms and rely on preventive drugs to safeguard their income, i.e. work. Relatable to income, the variable Financial distress reveals no explicit impact of difficulties with making ends meet on the use of preventive drugs. This suggests that respondents report no hindrance of financial difficulties to purchase preventive drugs for COVID-19 disease. The sole significant odds for this variable, provided by estimator (4), indicate that making ends meet fairly easily relates to higher odds of using preventive drugs. Yet, the remaining odds, as well as its interactions with Symptoms appear non-significant, implying that Financial distress is not statistically relevant for preventive drug intake. A more impactful variable is the Self-perceived health status, of which the worse conditions relate to a higher likelihood of preventive drug usage. Except for a single interaction effect, the impact occurs regardless of the respondent reporting symptoms or not. This single effect is reported by estimator (4) and reveals a boosted odds of using preventive drugs when the respondent reports having symptoms and being in a poor health status. Lastly, the two dimensions covering time and geography are tabulated. Estimator (3) reports higher odds of using preventive drugs in the second survey year, i.e. 2022. Yet, this effect is not confirmed by the same estimator with integrated longitudinal calibrated weights, implying that it may be irrelevant. Regarding the geographical dimension, two countries report an extremely high likelihood for preventive drug intake, i.e. Czech Republic and Estonia. Revisiting Figure 2, a single characteristic explains these extremes as both countries hold, relative to the other countries, a better balance between the Yes and No categories of reporting preventive drug intake. Soundly, this balance is picked up by the logistic estimators. The remaining countries with relatively high odds of reporting preventive drug intake for COVID-19 disease relative to the baseline Germany, are Switzerland, Belgium, Portugal, and Slovenia. No evident reason for the differences between countries is at hand. To conclude, the regression analysis highlights the importance of various drivers of preventive drug intake for COVID-19 disease across fifteen European countries.

## Discussion

This study documents an increased preventive drug intake when being female, more educated, with income, in worse health, and out of work due to COVID-19. In particular, those with symptoms report an additional increase in the odds of using preventive drugs when in poor health, with income, and when having hardly any difficulties with making ends meet. Surprisingly, increasing age while reporting symptoms results in lower odds of reporting preventive drug intake for COVID-19 disease. Relationship status and financial distress appear not to influence the preventive drug intake behaviour. Several countries indicate an increased likelihood of preventive drug usage, yet no explicit divide on the geographical level is found. In the context of the Behavioral Model of Health Services Use, the study reveals the influence of the Need component Symptoms on the Predisposing component Age, the Need component Self-perceived health status, and the Enabling components Income as well as Financial distress. Despite the study providing relevant insight, it comes with limitations. Firstly, the dependent variable covers preventive drug intake, yet does not distinguish self-medication with and without medical advice. As such, it may be regarded as a precautionary act, or an act influenced by a health worker or medical institution. This difference is relevant for health shock policymaking as the former implies stimulating drug intake, the latter seeking medical advice. Secondly, the preference for the random-effects estimators proposed in this study relies on a Hausman test, which requires additional theoretical support in the context of logistic panel estimators. Overall, the findings in this study may be regarded as valuable while acknowledging future research may provide improvements.

## Conclusion

By means of random-effects panel logistic as well as pooled logistic estimators, this study reveals how being female, out of work due to COVID-19, more educated, with income, or in worse health makes it more likely that preventive drugs are used for countering COVID-19 disease. Those who report symptoms are additionally more likely to show preventive behaviour when in poor health, with income, or having hardly any difficulties with making ends meet. The same stimulus of having symptoms appears to decrease preventive drug intake at older ages. Relationship status, financial distress, and geographical location are regarded as not relevant for preventive drug intake in this study. Overall, this study provides insight into preventive drug intake due to a health shock relevant for policymaking within the theoretical framework of the Behavioral Model of Health Services Use.

## Figures and Tables

**Table 1:** Descriptives

VARIABLES	Unique	Mean	Minimum	Maximum

Preventive drug intake COVID-19	2	-	0	1
Single or couple	2	0.624	0	1
Gender	2	1.615	1	2
Out of work due to COVID-19	3	-	0	2
Age	56	69.153	41	100
Years of education	29	12.462	0	25
Self-perceived health status	5	-	1	5
Reporting symptoms	2	-	0	1
Without or with income	2	-	0	1
Financial distress	4	-	1	4
Country of citizenship	15	-	12	55
Survey year	2	-	2020	2022

Number of observations: 4,649 for 3,111 respondents

**Table 2:** Logistic estimators explaining Preventive drug intake due to COVID-19 with Personal, Health and Partnership characteristics

VARIABLES	(1)	(2)	(3)	(4)

Reporting symptoms: No symptoms (base)				
Symptoms	2.172*** (0.399)	267.126** (626.094)	79.108** (145.841)	0.663 (2.373)
Relationship status: Single (base)				
Couple	0.968 (0.172)	0.989 (0.200)	1.010 (0.163)	0.838 (0.286)
Symptoms#Couple	-	0.907 (0.350)	0.944 (0.279)	0.916 (0.557)
Gender: Male (base)				
Female	1.671*** (0.306)	1.873*** (0.390)	1.694*** (0.279)	1.398 (0.471)
Symptoms#Gender	-	0.612 (0.240)	0.630 (0.193)	2.157 (1.208)
Age	0.986 (0.012)	0.996 (0.013)	0.996 (0.011)	0.991 (0.028)
Symptoms#Age	-	0.949** (0.024)	0.964* (0.019)	0.958 (0.038)
Years of education	1.058** (0.024)	1.064** (0.028)	1.051** (0.023)	1.014 (0.050)
Symptoms#Years of education	-	0.980 (0.041)	0.974 (0.032)	1.055 (0.067)
Out of work: Not applicable(base)				
Not out of work	0.900 (0.187)	0.938 (0.227)	0.988 (0.190)	0.730 (0.370)
Out of work	1.840* (0.587)	2.041* (0.774)	1.778* (0.546)	1.241 (0.767)
Symptoms#Not out of work	-	0.852 (0.382)	0.819 (0.288)	2.189 (1.583)
Symptoms#Out of work	-	0.574 (0.400)	0.572 (0.302)	0.622 (0.643)
Income: Without income (base) With income	0.417***	0.377***	0.923	0.456
	(0.066)	(0.069)	(0.233)	(0.239)
Symptoms#With income	-	1.710	1.569	4.543**
		(0.659)	(0.464)	(2.930)
Financial distress: With great difficulty				
With some difficulty	1.131 (0.503)	1.227 (0.648)	1.213 (0.503)	2.298 (1.969)
Fairly easily	0.974 (0.422)	1.216 (0.613)	1.284 (0.502)	8.799** (8.905)
Easily	0.772 (0.341)	1.005 (0.515)	1.112 (0.447)	4.453 (4.370)
Symptoms#With some difficulty	-	0.922 (0.832)	0.955 (0.669)	1.897 (3.029)
Symptoms#Fairly easily	-	0.582 (0.511)	0.641 (0.441)	1.238 (2.060)
Symptoms#Easily	-	0.458 (0.407)	0.548 (0.380)	0.575 (0.911)
Health status: Excellent (base)				
Very good	2.546** (0.973)	2.654** (1.232)	2.309** (0.884)	2.659 (2.214)
Good	2.865*** (1.066)	3.199** (1.463)	2.817*** (1.073)	2.122 (1.620)
Fair	2.994*** (1.178)	3.360** (1.607)	2.881*** (1.146)	2.794 (2.310)
Poor	2.857** (1.334)	2.644* (1.533)	3.026** (1.417)	0.787 (0.700)
Symptoms#Very good	-	0.843 (0.744)	0.809 (0.561)	3.387 (4.097)
Symptoms#Good	-	0.611 (0.523)	0.565 (0.380)	5.258 (5.999)
Symptoms#Fair	-	0.619 (0.559)	0.566 (0.398)	7.157 (8.651)
Symptoms#Poor	-	1.159 (1.209)	0.638 (0.511)	23.445** (33.026)
2020 (base)				
2022	-	-	2.020*** (0.476)	1.377 (0.708)
Germany (base)				
Sweden	1.017 (0.617)	1.006 (0.612)	0.949 (0.482)	2.020 (1.366)
Netherlands	1.806 (1.157)	1.816 (1.171)	1.597 (0.852)	2.962* (1.931)
Spain	2.472 (1.486)	2.456 (1.490)	2.000 (0.982)	2.071 (1.623)
Italy	1.991 (1.150)	2.037 (1.190)	1.727 (0.827)	2.337 (1.551)
France	1.591 (0.908)	1.536 (0.881)	1.436 (0.685)	2.516 (1.554)
Denmark	1.251 (0.803)	1.242 (0.804)	1.200 (0.660)	1.848 (1.294)
Switzerland	3.204** (1.842)	3.142** (1.822)	2.543** (1.205)	10.510*** (7.293)
Belgium	3.172** (1.649)	3.141** (1.648)	2.560** (1.103)	3.390** (1.981)
Czech Republic	28.551*** (17.386)	30.027*** (18.594)	12.774*** (5.993)	13.999*** (9.972)
Luxembourg	0.822 (0.579)	0.821 (0.583)	0.804 (0.477)	1.599 (1.663)
Portugal	3.224* (2.281)	3.347* (2.405)	2.667* (1.535)	6.430 (8.379)
Slovenia	4.308** (2.685)	4.225** (2.672)	3.096** (1.565)	6.155** (4.491)
Estonia	27.121*** (15.764)	27.324*** (16.060)	11.578*** (5.125)	19.045*** (11.164)
Finland	2.784 (1.852)	2.773 (1.849)	2.387 (1.285)	2.336 (1.643)
Constant	0.006*** (0.007)	0.002*** (0.003)	0.004*** (0.004)	0.003** (0.008)
Observations	4,642	4,642	4,642	4,585
Number of respondents	3,107	3,107	-	-
Panel structure	Yes	Yes	No	No
Effects	Random	Random	-	-
Standard errors (in parentheses)	Robust	Robust	Cluster	Cluster
Calibrated longitudinal weights	No	No	No	Yes

Calibrated longitudinal weights are provided by SHARE. The regressions rely on SHARE-data from Imputation 1. The estimation results are provided by logistic estimators which report odds ratios. Preventive drug intake due to COVID-19 is the dependent variable with 0 No and 1 Yes. Cluster indicates standard errors which account for the within respondent correlation. The Panel structure consists of the respondent’s unique identifier and the survey year. A hashtag indicates an interaction effect.

*** p<0.01,

** p<0.05,

* p<0.10

## Data Availability

To provide insight into the longitudinal analysis completed in this article, do- and log-files of Stata are available via https://github.com/hansgevers/preventivedrugintake.

## Appendix

Table 3 reveals the Spearman correlations between the variables referred to in Table 1.

**Table 3:** Spearman's rank correlation coefficients

	VARIABLES	1	2	3	4	5	6	7	8	9	10	11	12

1	Country of citizenship	1.000	-	-	-	-	-	-	-	-	-	-	-
2	Single or couple	−0.036	1.000	-	-	-	-	-	-	-	-	-	-
3	Gender	0.008	−0.205	1.000	-	-	-	-	-	-	-	-	-
4	Age	−0.117	−0.193	−0.002	1.000	-	-	-	-	-	-	-	-
5	Years of education	0.027	−0.002	−0.060	−0.185	1.000	-	-	-	-	-	-	-
6	Self-perceived health status	0.082	−0.126	0.081	0.181	−0.203	1.000	-	-	-	-	-	-
7	Reporting symptoms	0.051	−0.033	0.021	−0.054	0.025	0.050	1.000	-	-	-	-	-
8	Financial distress	−0.097	0.182	−0.120	−0.005	0.192	−0.269	−0.065	1.000	-	-	-	-
9	Preventive drug intake	0.147	−0.041	0.073	−0.024	0.033	0.073	0.089	−0.066	1.000	-	-	-
10	Survey year	0.015	−0.056	0.014	0.046	0.007	0.065	−0.003	−0.023	0.086	1.000	-	-
11	Out of work due to COVID-19	0.017	0.071	−0.026	−0.560	0.176	−0.188	0.042	0.031	0.030	0.091	1.000	-
12	Total household income*	−0.014	0.053	−0.026	−0.080	0.011	−0.072	0.011	0.003	−0.071	−0.897	−0.034	1.000

* base for the variable Without or with income

**Table 4:** Additional logistic estimators explaining Preventive drug intake due to COVID-19 with Personal, Health and Partnership characteristics

VARIABLES	(1)	(2)

Reporting symptoms: No symptoms (base)		
Symptoms	3.384*** (1.354)	2.172*** (0.403)
Relationship status: Single (base)		
Couple	0.000 (0.003)	0.968 (0.170)
Gender: Male (base)		
Female	-	1.671*** (0.305)
Age	3.712*** (1.861)	0.986 (0.012)
Years of education	-	1.058*** (0.022)
Out of work: Not applicable(base)		
Not out of work	0.347** (0.167)	0.900 (0.185)
Out of work	0.240 (0.254)	1.840* (0.603)
Income: Without income (base)		
With income	1.027 (0.566)	0.417*** (0.067)
Financial distress: With great difficulty		
With some difficulty	0.547 (0.462)	1.131 (0.495)
Fairly easily	0.351 (0.300)	0.974 (0.413)
Easily	0.195* (0.184)	0.772 (0.335)
Health status: Excellent (base)		
Very good	1.841 (1.740)	2.546** (1.017)
Good	1.311 (1.312)	2.865*** (1.107)
Fair	1.129 (1.205)	2.994*** (1.224)
Poor	0.948 (1.124)	2.857** (1.427)
Germany (base)		
Sweden	-	1.017 (0.639)
Netherlands	-	1.806 (1.223)
Spain	-	2.472 (1.549)
Italy	-	1.991 (1.214)
France	-	1.591 (0.925)
Denmark	-	1.251 (0.823)
Switzerland	-	3.204** (1.894)
Belgium	-	3.172** (1.705)
Czech Republic	-	28.551*** (18.508)
Luxembourg	-	0.822 (0.590)
Portugal	-	3.224 (2.420)
Slovenia	-	4.308** (2.796)
Estonia	-	27.121*** (16.134)
Finland	-	2.784 (1.872)
Constant	-	0.006*** (0.007)
Observations	342	4,642
Number of respondents	171	3,107
Panel structure	Yes	Yes
Effects	Fixed	Random
Calibrated longitudinal weights	No	No

Standard errors in parentheses. Calibrated longitudinal weights are provided by SHARE. The regressions rely on SHARE-data from Imputation 1. The estimation results are provided by logistic estimators which report odds ratios. Preventive drug intake due to COVID-19 is the dependent variable with 0 No and 1 Yes. The Panel structure consists of the respondent’s unique identifier and the survey year. The fixed-effects panel estimator in column 2 drops 2,936 respondents (i.e. 4,300 observations) as they consistently report the same value for the dependent variable over time.

*** p<0.01,

** p<0.05,

* p<0.10

## References

[R1] Agrawal A., Sharma A., Mathur M., Sharma A., Modi G., & Patel T. (2023). Perspective Toward Complementary & Alternative Medicines in the Prevention of COVID-19 Infection. Indian Journal of Community Medicine, 48(3), 401–406. 10.4103/ijcm.ijcm_282_22 37469923 PMC10353683

[R2] Ahmed I., Hasan M., Akter R., Sarkar B. K., Rahman M., Sarker M. S., & Samad M. A. (2020). Behavioral Preventive Measures and the Use of Medicines and Herbal Products Among the Public in Response to COVID-19 in Bangladesh: A Cross-Sectional Study. PLOS ONE, 15(12), e0243706. 10.1371/journal.pone.0243706 PMC7732085 33306725

[R3] Akintunde T. Y., Shaojun C., Oyeniran O. I., Etuh M. A., Tassang A. E., Chia T., & Amoo F. O. (2022). Personal Sensitivity, Self-Medication, Mask Usage, and COVID-19 Symptoms in Sub-Saharan Africans. Journal of Istanbul Faculty of Medicine, 85(2), 147–154. 10.26650/IUITFD.945803

[R4] Andersen R. (1968). A Behavioral Model of Families' Use of Health Services. Center for Health Administration Studies, University of Chicago.

[R5] Arain M. I., Shahnaz S., Anwar R., & Anwar K. (2021). Assessment of Self-medication Practices During COVID-19 Pandemic in Hyderabad and Karachi, Pakistan. Sudan Journal of Medical Sciences, 16(3), 347–354. 10.18502/sjms.v16i3.9696

[R6] Babitsch B., Gohl D., & von Lengerke T. (2012). Re-revisiting Andersen's Behavioral Model of Health Services Use: A Systematic Review of Studies from 1998–2011. Psychosoc Med, 9, Doc11. 10.3205/psm000089 PMC3488807 23133505

[R7] Benites-Meza J. K., Mejia-Bustamante A., Monzon-Monge D., Urrunaga-Pastor D., & Benites-Zapata V. A. (2022). Self-Medication in Peru During the COVID-19 Pandemic: How Harmless It Could Be? International Journal of Preventive Medicine, 13(1), 1–2. 10.4103/ijpvm.IJPVM_359_20 35706862 PMC9188890

[R8] Bergmann M., Wagner M., Yilmaz Y., Axt K., Kronschnabl J., Pettinicchi Y.,…Börsch-Supan, A. (2024). SHARE Corona Surveys: Study Profile. Longitudinal and Life Course Studies. 15 (4), 506–525. 10.1332/17579597Y2024D000000027 39370996

[R9] Börsch-Supan A., Brandt M., Hunkler C., Kneip T., Korbmacher J., Malter F.,…Zuber, S. (2013). Data Resource Profile: The Survey of Health, Ageing and Retirement in Europe (SHARE). International Journal of Epidemiology, 42(4), 992–1001. 10.1093/ije/dyt088 23778574 PMC3780997

[R10] Dare S. S., Eze E. D., Echoru I., Usman I. M., Ssempijja F., Bukenya E. E., & Ssebuufu R. (2022). Behavioural Response To Self-Medication Practice Before and During Covid-19 Pandemic in Western Uganda. Patient Preference & Adherence, 16, 2247–2257. 10.2147/PPA.S370954 36034331 PMC9400814

[R11] De Luca G., & Rossetti C. (2018). Survey of Health, Ageing and Retirement in Europe (SHARE) Computing Calibrated Weights. https://share-eric.eu/fileadmin/user_upload/pdf_documentation/SHARE_calibrated_weights_user_guide.pdf

[R12] ECDC (s.d.). Coronavirus Disease (COVID-19): Recommended vaccinations. European Centre for Disease Prevention and Control (ECDC). Retrieved July 2, 2026, from https://vaccine-schedule.ecdc.europa.eu/

[R13] FOPH (s.d.). COVID-19. Federal Office of Public Health (FOPH). Retrieved July 2, 2026, from https://www.bag.admin.ch/en/covid-19-en

[R14] Hashemi H., Ghareghani S., Nasimi N., & Shahbazi M. (2023). A Review of Poisonings Originating from Self-Administration of Common Preventative Substances During COVID-19 Pandemic. The American Journal of Emergency Medicine, 63, 147–148. 10.1016/j.ajem.2022.09.020 36175265 PMC9477789

[R15] Hausman J. A. (1978). Specification Tests in Econometrics. Econometrica, 46(6), 1251–1271. 10.2307/1913827

[R16] Jairoun A. A., Al-Hemyari S. S., Abdulla N. M., El-Dahiyat F., Jairoun M., Al-Tamimi S. K., & Babar Z.-U.-D. (2021). Online Medication Purchasing During the COVID-19 Pandemic: Potential Risks to Patient Safety and the Urgent Need to Develop More Rigorous Controls for Purchasing Online Medications, A Pilot Study from the United Arab Emirates. Journal of Pharmaceutical Policy & Practice, 14(1), 1–7. 10.1186/s40545-021-00324-9 33931118 PMC8086226

[R17] Mahmoudi H. (2022). Assessment of Knowledge, Attitudes, and Practice Regarding Antibiotic Self-Treatment Use Among COVID-19 Patients. GMS Hygiene & Infection Control, 17, 1–5. 10.3205/dgkh000415 PMC9284720 35909654

[R18] Martínez-De La Rosa W. Á., Rodríguez-Sanjuan A., Peláez-Cerpa M. J., Serrano-Torres J. S., González-Negrete R. A., Collazos-Lara Y. E., & Mendoza-Sánchez X. (2023). Self-medication in Medical Students During COVID-19 Pandemic. Duazary: Revista de la Facultad de Ciencias de la Salud, 20(1), 13–24. 10.21676/2389783X.5102

[R19] Mitjà O., & Clotet B. (2020). Use of Antiviral Drugs to Reduce COVID-19 Transmission. The Lancet Global Health, 8(5), e639–e640. 10.1016/S2214-109X(20)30114-5 32199468 PMC7104000

[R20] Moussa A. A., Omar F. D., Fiidow O. A., Ali F. H., & Babatunde S. M. (2023). Self-Medication Practices Against COVID-19 Infection and Awareness Among Residents of Mogadishu, Somalia: A Cross-Sectional Analysis. PLOS ONE, 18(6), 1–14. 10.1371/journal.pone.0284854 PMC10306195 37379300

[R21] Pacifico D. (2014). sreweight: A Stata Command to Reweight Survey Data to External Totals. The Stata Journal, 14(1), 4–21.

[R22] Pawar L., Srikanth, & C, S. S. (2023). Self-Medication Practice Among Medical Students in the Context of the COVID-19 Pandemic. International Journal of Nutrition, Pharmacology, Neurological Diseases, 13(3), 205–209. 10.4103/ijnpnd.ijnpnd_27_23

[R23] Pelullo C. P., Lombardi C., Della Polla G., Napolitano F., & Di Giuseppe G. (2025). Assessment of Knowledge, Attitudes and Behaviors Related to Self-Medication Practices Among the Adult Population During the COVID-19 Pandemic in Italy. Scientific Reports, 15(1), 1–10. 10.1038/s41598-025-22124-w 41174099 PMC12578919

[R24] Pimentel R. M., & de Souza M. W. (2024). Assessment of Medication Use in Students During the COVID-19 Pandemic. Cadernos ESP, 18(1), 1–11.

[R25] Quincho-Lopez A., Benites-Ibarra C. A., Hilario-Gomez M. M., Quijano-Escate R., & Taype-Rondan A. (2021). Self-Medication Practices to Prevent or Manage COVID-19: A Systematic Review. PLoS ONE, 16(11), 1–12. 10.1371/journal.pone.0259317 PMC8562851 34727126

[R26] SHARE-ERIC. (2024a). Survey of Health, Ageing and Retirement in Europe (SHARE) Wave 8. COVID-19 Survey 1 (Version 9.0.0). 10.6103/SHARE.w8ca.900

[R27] SHARE-ERIC. (2024b). Survey of Health, Ageing and Retirement in Europe (SHARE) Wave 9. COVID-19 Survey 2 (Version 9.0.0). 10.6103/SHARE.w9ca.900

